# Getting On in Gotham: The Midtown Manhattan Study and Putting the “Social” in Psychiatry

**DOI:** 10.1007/s11013-021-09751-4

**Published:** 2021-09-07

**Authors:** Matthew Smith

**Affiliations:** Centre for the Social History of Health and Healthcare, University of Strathclyde, Glasgow, UK

## Abstract

In the spring of 1962, a series of alarming headlines greeted American newspaper readers. From “New York Living for Nuts Only” and “One in Five Here Mentally Fit” to “Scratch a New Yorker, and What Do You Find?” and “City Gets Mental Test, Results are Real Crazy,” the stories highlighted the shocking and, to some, incredible statistics that fewer than one in five (18.5%) Manhattanites had good mental health. Approximately a quarter of them had such bad mental health that they were effectively incapacitated, often unable to work or function socially. The headlines were gleaned from *Mental Health in the Metropolis* (1962), the first major output of the Midtown Manhattan Study, a large-scale, interdisciplinary project that surveyed the mental health of 1660 white Upper East Side residents between the ages of 20 and 59. One of the most significant social psychiatry projects to emerge following the Second World War, the Midtown Manhattan Study endeavored to “test the general hypothesis that biosocial and sociocultural factors leave imprints on mental health which are discernible when viewed from the panoramic perspective provided by a large population.” Despite initial media and academic interest, however, the Midtown Manhattan Study’s findings were soon forgotten, as American psychiatry turned its focus to individual—rather than population—psychopathology, and turned to the brain—rather than the environment—for explanations. Relying on archival sources, contemporary medical and social scientific literature, and oral history interviews, this article explains why the Midtown Manhattan Study failed to become more influential, concluding that its emphasis on the role of social isolation and poverty in mental illness should be taken more seriously today.

## Introduction

In April 1962, alarming headlines flashed across US newspapers about mental illness in New York City. Entitled “New York Living for Nuts Only” and “Scratch a New Yorker, and What Do You Find?” the stories highlighted the shocking figure that 81.5% of Manhattanites had “‘mild, moderate, or impairing’ symptoms of mental disturbance” (Srole Papers, Box 1, Folder 209). Twenty-five percent were effectively incapacitated, often unable to work or function socially.^1^ What was going on in Gotham?

These stories were spurred by *Mental Health in the Metropolis: The Midtown Manhattan Study* (*MHM*), the first volume in the Thomas A. C. Rennie Series in Social Psychiatry. The Midtown Manhattan Study (Midtown) was a large-scale, interdisciplinary project that ran from 1952 to 1960 and investigated the relationship between socio-cultural factors and mental health in the Upper East Side of Manhattan. It was founded and initially directed by Cornell social psychiatrist Thomas A. C. Rennie (1904–1956) and funded by grants from the National Institute for Mental Health (NIMH), the Millbank Memorial Fund, the Rockefeller Foundation, the Grant Foundation, the Corporation Trust, the Lucius N. Littauer Foundation, and the Samuel Rubin Foundation.

Midtown was emblematic of social psychiatry, an approach to mental health that became highly influential after WWII. Although it has been associated with community mental health (Karno and Schwartz 1974), therapeutic communities (Jones 1962), and transcultural psychiatry (Opler 1956, 1967), during the post-war period, social psychiatry concentrated on identifying and addressing the socioeconomic causes of mental illness. According to Rennie, in introducing the inaugural issue of the *International Journal of Social Psychiatry* in 1955, social psychiatry was “a preventive psychiatry” intended to inform mental health policy. It was also an interdisciplinary approach, where psychiatrists teamed up with social scientists, chiefly sociologists, anthropologists, and psychologists. During its zenith, social psychiatry was highly influential. Most presidents of the American Psychiatric Association throughout the 1950s and 1960s described themselves as social psychiatrists and/or espoused its approach in their annual addresses. Moreover, as President Kennedy’s 1963 “Special Message to Congress on Mental Illness and Mental Retardation” indicates, social psychiatry’s preventive approach also reached the very heights of Washington, D.C. (Smith 2016).

Social psychiatry was rooted in the early twentieth century mental hygiene and child guidance movements, but was given a great impetus by the concerns about mental illness that emerged out of WWII (Grob 1991; Pols and Oak 2007; Scull 2011). A string of social psychiatry projects would be conducted during the 1950s, including August B. Hollingshead (1907–1980) and Frederick C. Redlich’s (1910–2004) study of New Haven (1958) and Alexander Leighton (1908–2007) and Dorothea Cross Leighton’s (1908–1989) Stirling County Study (Leighton 1959; Hughes et al. 1960; Leighton et al. 1963). Such studies were preceded by sociologists Robert E. L. Faris (1907–1998) and H. Warren Dunham’s (1906–1985) investigation of psychiatric epidemiology in Chicago during the 1930s (1939). They would provide the intellectual heft behind the community mental health movement, which culminated in the passage of the Community Mental Health Act of 1963 (Grob 1991; Sharfstein 2000).

Among all these studies, Midtown was the most ambitious, due to its size, complexity, scope, interdisciplinarity, and novelty. In addition to over a dozen senior investigators, the study utilized twenty-three pre-doctoral research aides, twenty-three social scientists and social workers, and ninety-nine volunteers (fourteen of whom contributed over 500 h). It 1987, *MHM* was named a ‘Citation Classic’ by the Institute of Scientific Information, indicating its ongoing significance (American Sociological Association 1988). The American Sociological Association noted that while the study had been “[g]reeted by a storm of controversy, it was also “a milestone of social research” (10). It introduced novel methodologies, that would become standard practice, including probability sampling, the use of standardized forms, and employing machine technology.^2^ Unlike previous studies, it used not only data from psychiatric hospital admissions, but also sampled the mental health of an entire community.

Despite its significance, however, Midtown has not been extensively researched. The late Gerald Grob’s magisterial account of post-war American mental health policy, for example, describes Midtown as “famous,” but only provides a thumbnail sketch of it and its findings (1991:101). Others writing about post-war US psychiatry have not discussed it at all or only mentioned it in passing (Shorter 1997; Staub 2011; Torrey 2014). The most extensive descriptions of Midtown is found in the work of psychiatry professor Dan Blazer in *Age of Melancholy* (2005), and articles by historian Hans Pols (2003) and by epidemiologist Dana March and historian Gerald M. Oppenheimer (2014). By examining Midtown in depth, I hope to assert the contemporary importance of reappraising social psychiatry and its focus on prevention, as well as the value in delving deeply into the internal histories of such studies to understand their legacy and ongoing relevance.^3^

This is because, just as historians have overlooked Midtown, psychiatrists have also largely forgotten it. While *MHM* was widely reviewed in psychiatry journals, by the 1970s, psychiatrists had turned their focus to individual—rather than population—psychopathology, and to the brain—rather than the environment—for explanations.^4^ Examining the broader context of US psychiatry and politics during the late 1960s and 1970s, it is possible to identify many reasons why social psychiatry waned (Smith 2016). In Washington, D.C., politicians, such as Secretary of Health, Education, and Welfare John W. Gardner (1912–2002), were predicting a “crunch between expectations and resources” with respect to the sort of social programs that could have followed from the findings of social psychiatrists (1968). Social psychiatry was also experiencing a “crunch.” Initial optimism in psychiatry’s ability to prevent mental illness during the post-war period had also faded. In its place emerged a resurgent biological psychiatry, on the one side, and an antipsychiatry movement that questioned the notion of mental illness altogether, on the other. Community mental health centers, meant to be preventative, ended up treating the thousands of deinstitutionalized patients released from asylums (Kritsotaki, Long, and Smith 2016). The prevention of mental illness, the driving ambition of social psychiatry, was no longer prioritized.

But factors connected to the history of Midtown itself also illuminate how the message of social psychiatry could be undermined. In order to understand the demise of social psychiatry, therefore, it is also important to delve deeply into how prominent social psychiatry projects, such as Midtown, were designed and conducted, and how their findings were interpreted and articulated. Using archival sources, publications related to Midtown, and oral history interviews, I argue that Midtown’s legacy was hampered by tragedy, in-fighting about how to keep the interdisciplinary project unified, and hesitancy and division with respect to its conclusions. Given that Midtown had the potential to be the most influential of all the post-war social psychiatry studies, these issues dampened the impact, longevity, and significance of social psychiatry as a whole. This provides all the more reason to reconsider the preventive message of *MHM* amidst the mental health crises of today.

## Introducing Midtown

Midtown intended to “test the general hypothesis that biosocial and sociocultural factors leave imprints on mental health” (Srole et al. 1962:31).^5^ It did so by combining the insights and approaches of psychiatry, epidemiology, and social science research. This plan was envisioned by Thomas Rennie, who founded the project and directed it until his death in 1956. Born in Motherwell, Scotland, Rennie immigrated to Pittsburgh in 1910. After graduating from the University of Pittsburgh and Harvard Medical School, Rennie spent 1931–1941 at Baltimore’s Henry Phipps Psychiatric Clinic, where he worked with Adolf Meyer (1866–1950), one of the most influential contemporary psychiatrists. Rennie espoused Meyer’s concept of psychobiology, which described how mental disorder was due to an adaptive personality disorder, not a diseased brain, and that psychiatrists had to consider the biological, psychological, and social context of the patient. For Rennie “the principles of psychobiology were simple: to determine why and how the patient’s mental disorder developed” (Lamb 2014:98). Essentially, this was the ultimate goal of Midtown. Rennie also followed Meyer in his respect for the social sciences. He left the Phipps Clinic in 1941 for Cornell, where in 1950, he became the first US Professor of Social Psychiatry.

Rennie recruited a Midtown project team consisting of both mental health professionals and social scientists. The interdisciplinary nature of the project was mirrored in both the mix of and the positions held by the core members, including: Leo Srole (1908–1993), Professor of Sociology and Psychiatry at SUNY Medical Center; Thomas S. Langner, Assistant Professor of Sociology (Psychiatry) at Cornell; Stanley T. Michael, Research Associate in Psychiatry at Columbia; Marvin K. Opler (1914–1981), Professor of Social Psychiatry at the University of Buffalo School of Medicine (previously at Cornell); and Leighton, Professor of Sociology and Anthropology at Cornell and head of the Cornell Program in Social Psychiatry. The core team was also supported by psychiatric social workers, clinical psychologists, psychiatrists, and anthropologists Eleanor Leacock (1922–1987) and Vera Rubin (1911–1985), who worked on the project from 1952–1955 and 1953–1955, respectively.

The “keystone” of Midtown was a home interview survey (HES) of 1660 residents of Yorkville, a neighborhood of approximately 175,000 people (in 1954) on Manhattan’s Upper East Side (Srole et al. 1962:28). As Sarah Igo has shown, surveys, such as Robert Lynd (1892–1970) and Helen Lynd’s (1896–1982) Middleton studies, became an authoritative way of producing “objective” knowledge of particular groups of people through “observation, intensive fact-collecting, and quantification” (2007:28, 66). A similar study to that of Middleton was W. Lloyd Warner’s (1898–1970) “Yankee City” study, to which Srole contributed (Ira Srole Interview 2016). These strategies would be taken up by the Midtown researchers.

The HES was paralleled by an “Ethnic Family Operation” (EFO) led by Opler and intended to form the basis of the third Midtown volume (Srole et al. 1962:284). It was also supplemented by a “community sociography,” which accounted for the broader social and historical context of Midtown, and a “treatment census,” which provided a snapshot of the 2240 Yorkville residents currently undergoing psychiatric treatment (Srole et al. 1962:28). While the treatment census provided data on *treated* mental illness, Midtown—unlike most other social psychiatry studies—also investigated the mental health of the entire community, the suggestion being that those in treatment merely represented “the visible portion of an iceberg” (Srole et al. 1962:31).

The HES explored what lay beneath the surface, focusing on a sample of white residents between the ages of 20 and 59.^6^ One element of the survey was an interview that lasted approximately two hours, and was designed to be “less fluid than a psychotherapy session,” but “more freewheeling” than a medical intake interview (Srole et al. 1962:38). Its questions were designed to tease out particular psychiatric symptoms, including anxiety or depression. For example, respondents were asked to agree or disagree to the following statements: “You sometimes can’t help wondering whether anything is worthwhile anymore;” “I am often bothered by nervousness (irritable, fidgety, tense);” or “would you say most of the time you are in low spirits?”(Srole Papers, Box 56). Interviewers would probe further if the answer was “don’t know” or “no response” (Srole Papers, Box 60). These probes were anticipated to provoke “free association elaborations and asides” that would provide further insights into the interviewee’s mental health (Srole et al. 1962:40).

The interviewers also made a note of the interviewee’s behavior, including whether they were “hostile,” “suspicious,” “friendly,” or “solicitous,” about being interviewed, as well as overall impressions about them and their homes (Srole et al. 1962:390). Interviewers observed interviewee’s “apparent ease or tension, affect or mood, appropriateness of replies, apparent level of intelligence, dress and grooming habits, the presence or absence of muscular tics, stutter or stammer in speech, memory difficulties, and physical deviations and disabilities” (Srole et al. 1962:40–41) Such observations were described as roughly “parallel” to the practices employed by “the trained administer of a projective test like the Rorschach” (Srole et al. 1962:38), which Rebecca Lemov has explored (2015). One interviewer reported that: “Poverty, ignorance and neglect are the most striking features of R’s home,” adding that “R” might drink ten beers in a tavern with friends on payday.” Overall, the interviewer found “R” “contemptuous and suspicious” with the “boastful charm of an adolescent boy” (Srole Papers, Box 56).

Such factors, along with data about previous psychiatric and social services involvement, would help two of the project’s psychiatrists, Kirkpatrick and Michael, rate each respondent’s mental health. The specific ratings were broken down accordingly: well; mild symptoms; moderate symptoms; and impaired, which was further broken down into marked symptoms; severe symptoms; and incapacitated. Once the demographic details about the respondents (including age, marital status, ethnicity, socioeconomic status (SES), religion, and rural–urban background) were mapped onto these ratings, the researchers could delineate the relationship between these factors and mental health. These approaches were indebted to previous efforts to map SES and other factors, such as heredity, to mental health. Theodore M. Porter has shown how statistics pertaining to asylum patients from a range of countries dating to the early nineteenth century helped to associate a link between mental disorder and heredity (2018). Associating SES with mental health was also a feature of the mental hygiene and child guidance movements of the early twentieth century (March and Oppenheimer 2014).

Ultimately, the key finding of Midtown was that SES heavily influenced mental health: “Offspring of low SES-origin families at all age levels reflect maximum vulnerability to mental morbidity and minimum fulfillment of wellness” (Srole et al. 1962:356). The gap between low and high SES was particularly striking with respect to the ‘well’ and “impaired” categories. Whereas 30% of individuals currently in the highest SES strata were considered “well,” only 4.6% of those in the lowest SES strata were (Srole et al. 1962:230). Similarly, while 12.5% of those in the highest strata were considered “impaired” (with 0% “incapacitated”), 47.3% of those in the lowest strata were considered “impaired” (with 9.3% “incapacitated”). These findings echoed those of other social psychiatry studies, which emphasized the negative impact poverty, inequality, and social isolation had on mental health. Establishing findings, however, was one thing; interpreting them and informing mental health policy was something else.

## Initial Tensions

Any project of the size, complexity, novelty, and ambition of Midtown was bound to encounter problems. Yet, archival evidence reveals an array of upheavals, rivalries, and interdisciplinary divisions that undermined its impact. Such factors demonstrate that, while some of Midtown’s problems were merely down to misfortune, underlying tensions also existed regarding to how to conduct social psychiatry studies. These challenges distracted Midtown’s researchers from the ultimate aim of the project, which was to develop preventive approaches to psychiatry. Energy that could have been spent on translating their groundbreaking research findings into preventive mental health strategies were instead devoted to debates and disagreements. The acknowledgement of these difficulties, in turn, casts the legacy of Midtown and that of social psychiatry more broadly in a different light. Rather than dismissing the contributions of social psychiatry as overly idealistic or fanciful because they did not result immediately in better mental health policy, as Grob and Torrey have done, it becomes possible to interpret them more generously. It is better to see the ideas underlying social psychiatry not as inherently flawed, but rather as having unfulfilled potential or representing a missed opportunity. Thinking about the history of social psychiatry and its message about SES and mental illness in this way can, in turn, spur us to reconsider how we might tackle the social determinants of mental health today.

Many of Midtown’s problems can be traced to the untimely death of Rennie in 1956. However, an earlier argument between the project’s anthropologists foreshadowed the issues that would follow. The dispute primarily involved anthropologist Marvin Opler, and anthropologists Eleanor Leacock and Vera Rubin. Opler had invited Leacock and Rubin onto the project, having met them at Columbia (Opler Papers, Box 13, Folder 18). Both women would go onto prominence in their field. Leacock became well known as a Marxist-feminist cultural anthropologist and would eventually chair the CUNY’s anthropology department. She was made EFO Assistant Director in 1952.^7^ Rubin specialized in Caribbean studies, aging, and medical anthropology. She founded the Research Institute for the Study of Man in 1955 and co-founded the journal *Transcultural Psychiatry*. She became the other EFO Assistant Director in 1953.

The conflict ignited after Opler sent out a memo to all anthropology staff on September 24, 1954, whereby he set out five “rules… made effective here and now” to ensure that the next phase of field work “be productive and efficient, and that professional ethics, manners and careers be preserved and promoted to best effect in the future.” (Opler Papers, Box 14, Folder 8). Opler concluded:

We all face large tasks … and probably some difficulties in accomplishing goals of research. With efficient and mature judgment, and a modicum of trust and considered exchange of opinion, we can also have some real success in research. As an anthropologist I hope we can make good use of an opportunity, developed in medical science, to benefit human beings. Being also, like you, in the same profession and biological species, I will appreciate any attempts to reach consensus (Opler Papers, Box 14, Folder 8, emphasis in original).

This appeal was not successful. Rubin and Leacock responded with their own memo, which has not been preserved in the archives, but, according to Opler, took the form of an ultimatum.^8^ Opler immediately wrote to Rennie promising to meet with his fellow anthropologists and convince them to “fully and wholeheartedly accede to one Director of the Study” and “one in charge and supervision [sic] of all anthropology work” (Opler Papers, Box 13, Folder 17, emphasis in original). Rennie followed up on October 4, 1954, clarifying the project’s line management (Opler Papers, Box 13, Folder 17). Soon, however, the situation was deteriorating again. Opler told Rennie that, while he had “solved admirably a then-current crisis carried to some lengths by two of my colleagues [Rubin and Leacock],” it had led to “more work, less accomplished, and continual clash, with many of his colleagues “acting out” in meetings (Opler Papers, Box 13, Folder 17). Those “acting out” included Srole, foreshadowing disputes to come.

The situation worsened. In January 1955, Opler explained that he had taken up Rennie’s suggestion to “effect rapport” with Leacock, asking her “at her desk and with warmth and concern if we could discuss any matters she might have on her mind.” Leacock did not respond positively and, when he tried again later, she left the office “with something of a tirade including, ‘I don’t see that I have anything to discuss with you.’” Opler was convinced that Rubin and Leacock “had attempted, in effect, a serious and self-interested ‘strike’ against research,” “had damaged morale,” and “had gone into high gear to tear a carefully built structure down.” The team was at an impasse, leaving Opler to conclude that “Old Humpty Dumpty cannot be put back together again after 4 months of irresponsible functioning.”

Rubin and Leacock further demanded that the anthropology projects be split into two, independent studies with separate teams and publications. For Opler, this was anathema. He was adamant that Midtown had to be conducted as one coherent project, and that its findings had to be articulated with one voice, as was Rennie’s vision. If this was proving difficult among a team of anthropologists, it did not bode well for the interdisciplinary collaborations to come. Opler wrote again to Rennie on February 18, 1955, stating that he was “embarrassed by the fact that both [Rubin and Leacock] are anthropologists,” calling them as “hostile” and “unethical.” (Opler Papers, Box 13, Folder 18). Later he would apologize to Rennie for recommending Rubin and Leacock, describing them as “basically hostile individuals,” who were “shallow and self-interested,” and ultimately suggesting that they be fired (Opler Papers, Box 13, Folder 18).

The records do not show, however, whether Rubin and Leacock were fired or whether they left voluntarily after the completion of the EFO in June 1955. What they do indicate is how large-scale social psychiatry projects, such as Midtown, were not only idealistic attempts to determine the causes of mental illness and take steps to prevent them. They were also opportunities for “empire building, control of others, and STATUS” (Opler Papers, Box 13, Folder 19; emphasis in original). Putting career ambitions aside, however, the conflict highlighted divisions about how cohesive social psychiatry projects should be. While Rennie and Opler insisted that the integration of Midtown’s various parts was essential, others were less convinced.

Opler’s stubbornness about Rennie’s vision was related to his devotion to Rennie himself, who had effectively saved his career. Opler had graduated with a PhD from Columbia in 1938, having conducted research on the Ute and Paiute tribes in Colorado and Utah under the supervision of pioneering anthropologist and folklorist Ruth Benedict (1887–1948). Opler would find a position that year at Reed College in Oregon, where he became interested in psychiatry. In 1943, however, he was appointed to the War Labor Board. According to historian David H. Price (2004), the FBI was informed that Opler had previously been involved in (to them) suspicious left-wing activities. Opler was soon assigned to a Japanese-American internment camp at Tule Lake, Oregon to work as its chief community analyst (Suzuki 1981). The FBI continued to investigate him, taking note of his friendly relations with the internees. Although Price notes that a final interview with Opler in December 1945 was the end of the FBI’s interest, an oral history interview with Opler’s son, Lewis Opler (1948–2018), reveals otherwise.

After the war, Opler was awarded a Fulbright to do research in Burma and was offered an endowed chair at Harvard. Then, disaster struck. First, the funding for the Harvard position evaporated and then the FBI told Opler that he would not be able to get a visa for Burma unless he spied for them. Refusing, Opler found himself unemployed. After taking on some temporary positions Opler relied on his prior experience researching patients at Morningside Hospital in Oregon, and began applying for positions in academic psychiatry (Opler 1956). He wrote 88 letters to psychiatry departments, including one to Rennie at Cornell, who was planning Midtown. Despite knowing that he was blacklisted, Rennie hired him.^9^ Lewis Opler described how his father believed Rennie “saved” his family and how Opler “loved Rennie,” and was “very supportive” of him, sentiments which help explain why he was so determined to ensure that Rennie’s vision came to fruition.

## Replacing Rennie

Rennie died of a cerebral hemorrhage on May 21, 1956. His replacement was Alexander Leighton, who was already directing the Stirling County Study.^10^ Leighton had also studied under Adolph Meyer and, holding qualifications in both psychiatry and anthropology, he truly embodied social psychiatry’s interdisciplinarity (Tremblay 2009).^11^ But his focus was on Stirling County, not Midtown. Indeed, Leighton’s statement in *MHM*’s foreword that Rennie’s death was “a loss from which the Midtown Manhattan Project could not fully recover” was truer than most readers realized (Srole et al. 1962:viii). According to Lewis Opler, Cornell had never provided sufficient support for Midtown. Rennie was not given research leave to direct the project and did so on his own time. When Leighton took over, his mission was effectively to “wind it down,” despite the fact he was “heavily beset with his own … responsibilities” (Opler interview; Srole et al. 1962:337). For instance, he would spend 1957/1958 in Stanford writing one of his Stirling County outputs and not managing Midtown in person (Leighton Papers, Box 50, Folder 3). Although Midtown’s data had largely been collected by then, the task of analyzing and publishing its findings was only beginning. This difficult process would further disrupt an already divided team.

Opler and Leighton did not have a warm relationship. One source of Opler’s coolness was Leighton’s review of Opler’s *Culture, Society, and Human Values* (1956) in *Social Problems*, which was published a few months after Rennie’s death (Leighton 1956).^12^ In a letter expressing his frustration at Midtown’s progress, Opler described the contrast between Leighton’s review in his book and that of Harvard anthropologist Clyde Cluckhohn (1905–1960), describing them “as different as night and day” (Opler Papers, Box 13, Folder 12; Cluckhohn 1957).^13^ Opler “wondered if there is anything about me which suggests, even remotely, threat, deceit, formalism or lack of concern about your own valid interests and accomplishments, past, present, or future.” After Opler left Cornell in 1958, the two sparred over the need to obtain departmental clearance for publications coming out of the Midtown research and other matters (Opler Papers, Box 13, Folder 12; Box 13, Folder 3).

There was even more animosity between Opler and Srole. Rennie was generally successful in managing these two strong personalities or, as Lewis Opler stated, he at least “kept everybody from destroying each other.” Leighton was not so successful. Srole and Opler’s mutual antipathy masked similar backgrounds. Both emerged from relatively modest Jewish upbringings to excel academically. Srole’s mother died when he was an infant and he spent time in foster homes prior to his father remarrying, an experience that made him “a very sensitive guy” (Srole interview). Shy and withdrawn during high school, Srole would “force himself out of his shell,” and join the school debating team. After completing undergraduate studies at Harvard, he proceeded straight into doctoral studies at the University of Chicago. After some teaching experiences in New York, Srole became a military psychologist with the US Air Force in 1943, and then the United Nations Relief and Rehabilitation Administration Welfare Director of Landsberg Jewish Displaced Persons Camp. He resigned in 1945 in protest at the poor conditions there. His resignation covered in the *New York Times,* Srole would go onto lecture about his experience, being heralded as “the portent of the new generation of leadership arising in American Jewry” (Srole Papers, Box 1, Folder 22a). Srole’s son, Ira Srole, also described how his father managed to extract some of his family members who had been exiled by the Soviets to Siberia (Srole interview). Back in the USA, Srole worked for the Anti-Defamation League before joining Midtown in 1952 (Srole interview). These experiences shaped Srole—much like Opler—into a forthright, yet sensitive, individual who had the willingness and ability to debate his views vigorously. According to his son—and similarly to Opler—Srole had “a fairly quick temper,” “did not suffer fools gladly,” was “not to be trifled with,” and “was not at all shy about expressing his opinions. If he needed to make waves, well, so be it. He made waves.”

The bitterness between Opler and Srole was often expressed in relation to the content and authorship of Midtown’s outputs. The ghost of Rennie loomed large. On March 27, 1957, Leighton attempted to settle the issue, proposing that the project be divided into two teams: one led by Srole (supported by Langner) and focused on completing volumes I and II, and one led by Opler and focused on completing volume III (Opler Papers, Box 13, Folder 12). Although Opler agreed to this division, he warned it might undermine the volumes’ “interdisciplinary strengths” and add to the “divisiveness and partial analysis that I have deplored,” resulting in an “inferior product” that would “fail to deliver the kind of scientific products for which we are responsible,” thus “damaging” the reputations of all concerned, including the deceased Rennie (Opler Papers, Box 13, Folder 12). For Opler, “inferior” meant a project that was a loose conglomeration of related studies, rather than a coherent whole that could inform mental health policy. In a marginal note, Opler reminded Leighton that: “Obviously, I am as concerned in March, 1957, about what my or Rennie’s name is attached to, as I was in June, 1952, or will be in January, 1958.”

A few months later, Opler announced that he would be leaving Cornell for the University of Buffalo in May 1958. The debates, however, would continue. One thorny issue concerned the volumes’ authorship, which emerged in June 1960, when Srole and Langner consulted legal advice to change the original publication contract. Writing to McGraw-Hill, Srole argued that Rennie’s death had “changed matters drastically” (Opler Papers, Box 13, Folder 14). Rennie had only written one chapter, Srole contended, and, since he had not edited the volumes, it could not be assumed that he “would have accepted responsibility for the whole structure of assumptions, formulations, interpretations, and hypotheses that each volume has erected.” He proposed that, while the series be named in Rennie’s honor, Rennie should not be listed as an author of Volumes II–III, and that Opler’s name should not be listed for Volume I.

Opler mulled over his response in a letter to his elder brother, the anthropologist Morris Opler (1907–1996). Although he was outraged, he could not afford a legal battle, and asked his brother for his advice “before plunging” (Opler Papers, Box 13, Folder 14). He also corresponded with Rennie’s brother concerning the matter. Correspondence between the various parties, would continue until November, 1960, when authorship was finally agreed: Opler’s name would be added to Volume I, but Rennie’s name would not appear on Volumes II–III.^14^

These disputes attenuated Midtown and its legacy. What were meant to be three volumes in the Thomas A. C. Rennie series became two. The third, to be completed by Opler, was drafted, but never published. The second, *Life Stress and Mental Health*, was originally meant to be authored by Srole, Langner, and Michael, but Srole’s name was not ultimately listed (Langner and Michael 1963). It was published in 1963 within days of President Kennedy’s assassination, and failed to receive its predecessor’s media coverage (Srole Papers, Box 2, Folder 43).^15^ Srole would, however, publish two follow-up studies: the first was written with Anita K. Fischer in 1978; the second was published posthumously with psychiatrist Ernest Joel Millman in 1998. Neither would garner the excitement that attended *MHM*.

## Midtown’s Message

In introducing the “Goals and Guidelines of the Study,” Srole described how Midtown was intended to derive both “*basic research* … about the still elusive mainsprings of most mental disorder processes,” and “*program research* to guide public and professional policy” (Srole et al. 1962:8–9, emphasis in original). One might have expected that Midtown’s key finding about SES and mental health would have easily translated in to such “program research” and policy recommendations. Instead, the bulk of *MHM* focused on “basic research,” and even here, its conclusions tended to be presented in a “restrained,” tentative manner (Srole et al. 1962:340).

This restraint was due in large part to what Srole described as “the Tower of Babel potentialities in an interdisciplinary team with different scientific approaches and degrees” (Box 13, Folder 17, Opler Papers). In a 1967 interview, Srole described how “only selected investigators have the personality for multidisciplinary research” (Box 1, Folder 33d, Srole Papers). Such interdisciplinary divisions were exemplified by *MHM*’s two “Epilogues”—one written by psychiatrist Stanley Michael and one written by Srole. Having two conclusions already went against Rennie and Opler’s vision for a unified project, but the content of the “Epilogues” revealed even more gaps. Michael began his “Epilogue” by stating that *MHM* was “written by sociologists … based on sociological data,” thus downplaying the role of psychiatrists, such as himself (Srole et al. 1962:327). Further, “the reader … may be impressed with a sense of sociogenic overdeterminism. This impression is further strengthened by the fact that manuscript was written by sociologists” (Srole et al. 1962:328). Michael also stressed that cautious application of Midtown’s findings was necessary, thus undermining the goal of “program research.” Although he concluded by stating that Midtown would be “a springboard for future research in social psychiatry,” his “Psychiatrist’s Commentary” did not amount to a ringing endorsement (Srole et al. 1962:335).

Srole also qualified Midtown’s findings, suggesting that they would only serve as “temporary scaffolding for constructing new and more focused hypotheses” (Srole et al. 1962:342). His policy recommendations were articulated obliquely and were restricted to initiatives, such as parent education, which had long been established (Grant 1998; Jones 1999; Bakker 2020).^16^ Srole later admitted that “investigators who conduct a large scale undertaking like the Midtown Study, incorporate in their scientific roles neither responsibilities nor skills in conveying their findings to the lay community or in implementing their findings on the social policy level” (Srole Papers, Box 1, Folder 69). According to Srole, who viewed Midtown’s impact as “imperceptible,” this “split between study and action” was unfortunate, and that funders should support such “diffusion of knowledge.” Srole seemed to be suggesting that, with sufficient funding, perhaps the policy implications of Midtown would have been better articulated. Ultimately, however, it was only in Chapter 12’s conclusion, co-written by Srole and Langner, where any hint of policy implications was expressed:

interventions into the downward spiral of compounded tragedy, wherein those handicapped in personality or social assets from childhood on are trapped as adults at or near the poverty level, there to find themselves enmeshed in a web of burdens that tend to precipitate (or intensify) mental and somatic morbidity; in turn, such precipitations propel the descent deeper into chronic, personality-crushing indigency (Srole et al. 1962:236).

Among the publications that would emerge out of Midtown, this flowery statement came closest to detailing what had to be done to prevent mental illness, the guiding mission of social psychiatry. It left the reading guessing, however, about what the “interventions” should be.

## Conclusion: Midtown Today

This article has not intended to speculate “what might have been” had Midtown been undertaken in a more harmonious environment. Social psychiatry enjoyed a remarkable amount of influence during the post-war period, but waned during the 1970s and 1980s in the face of a host of internal and external pressures. While Midtown benefitted from the initial enthusiasm in social psychiatry, its impact also declined in parallel. Ultimately, Rennie’s ambition for “psychiatry to move into a vigorous period of real preventive work” was not realized (Srole et al. 1962:5–6). The article has argued, however, that, in order to understand the legacy of Midtown—and social psychiatry more generally—it is necessary to investigate *how* the study was produced, as well as *what* it produced. Thus informed, we can consider the relevance of social psychiatry today.

And it is clear that social psychiatry, despite being ignored and forgotten, could hardly be more relevant in 2021. This article has been written in the midst of the COVID-19 pandemic and subsequent lockdown. One of the pandemic’s many seismic effects has been its impact on mental health. While many are justifiably anxious about the threat of the virus itself, the mental health of others have suffered from the social isolation, loneliness, and financial insecurity incurred by lockdown measures (Xiong et al. 2020). The mental health of people marginalized on the basis of race, ethnicity, or SES has been even more affected by COVID-19 (McKnight-Eily et al. 2021; Nagata et al. 2021). None of this would have surprised the Midtown researchers. Social isolation, poverty, and inequality were all factors that loomed large in *MHM*, as well as other social psychiatry studies. The question, now as then, is what to do about it. While many were arguing before COVID-19 that the USA was in the midst of a mental health crisis (Plakun 2020), the pandemic may have provided the impetus for the sort of bold socioeconomic initiatives that could have emerged from the heyday of social psychiatry, but did not. One such intervention that has received additional attention during the pandemic is universal basic income (UBI), an unconditional and guaranteed income provided to all individuals (Haagh 2019; Nettle et al. 2021). Indeed, many countries, including the USA, have introduced COVID-19 income support schemes that bear some similarity to UBI (US Congress 2020). The pandemic has also spurred some health experts to suggest that a UBI might be one way to achieve economic recovery and improve health outcomes (Patel and Kariel 2021).

A version of UBI was recommended in one of the few documents published during the zenith of social psychiatry that articulated a series of clear preventive mental health strategies. In 1969, the Joint Commission on Mental Health of Children published *Crisis in Child Mental Health: Challenge for the 1970s*. One of the measures that the authors urged the Federal Government to implement was a guaranteed minimum income, along with a national health service and a guaranteed employment program. Needless to say—and fifty years later—these measures were not adopted. But should they be considered today? Could UBI improve mental health and reduce mental illness? There have been some studies investigating the relationship between guaranteed incomes of various kinds (including UBI pilots) and mental health. Evelyn Forget, for instance, found that the minimum income experiment in Dauphin, Manitoba during 1974–1979 (MINCOME) resulted in fewer hospitalizations for mental illness (2011). Research has also shown, for example, that family supplements (from casino revenue) paid to Native Americans in the Great Smoky Mountains resulted in lower rates of mental illness in young adults (Costello et al. 2010). But many other UBI pilots have not measured mental health explicitly. Revisiting the history of social psychiatry, therefore, can provide the impetus to consider the link between SES and mental illness more carefully and, in particular, explore the potential of interventions, such as UBI.

Midtown and other social psychiatric studies found that mental illness was associated with poverty, inequality, social isolation, and community disintegration (Faris and Dunham 1939; Hollingshead and Redlich 1958; Leighton 1959, Hughes et al. 1960; Leighton et al. 1963). These findings have been supported by subsequent studies, though most of the solutions offered have failed to address the structural problems contributing to such socioeconomic problems (Sørensen et al. 2013; Matthews et al. 2015; Blair and Raver 2016; Wilkinson and Pickett 2018). UBI could be one way of mitigating many of these factors. If set at a sufficient rate, it would lift individuals out of poverty, including those working in caring capacities without pay. The fact that UBI is guaranteed would alleviate much of the stress associated when welfare and unemployment benefits fluctuate (Moffatt et al. 2016). It might also help provide people trapped in abusive domestic situations the financial wherewithal to leave and access support. UBI would help address inequalities by making it easier for people to attain higher education at different life stages, pursue entrepreneurial and creative enterprises, and transition from one career path to another. By providing an income to volunteers, social activists, and community organizers, it could also help to reduce social isolation and build community cohesion, factors that the Stirling County Study particularly identified. It is worth adding that a UBI would also be a great support to those already dealing with mental health problems. In addition to improving mental health in the short term, a UBI could also help to reduce the socioeconomic factors that contribute to Adverse Childhood Experiences (Akee et al. 2018; Lacey et al. 2020). UBI would certainly not be a panacea, but it could provide the foundation for less poverty, inequality, and social isolation, all factors that either contribute to or exacerbate mental illness.

The increased interest in UBI during the pandemic, as well as the provision of temporary income schemes, might well result in its implementation in the near future somewhere in the world. There are also a growing number of pilots, including some based in the USA, and UBI has been increasingly endorsed by political parties, such as the Green Party, politicians, such as the former Democratic presidential candidate and New York City mayoral candidate Andrew Yang, and even entrepreneurs, such as Twitter founder Jack Dorsey. Nevertheless, there also continues to be significant resistance to the idea. Regardless of whether UBI becomes more of a mainstream policy or not, the point is that now is time to seriously consider the sort of progressive socioeconomic changes that could benefit mental health and prevent mental illness. This article has explained why Midtown failed to achieve more significance than it did, but perhaps if we learn more about its history and that of social psychiatry more generally, it can still influence the development of a truly preventive approach to mental health today.

## Footnotes

1 In contrast, Thomas Szasz had published *The Myth of Mental Illness* in the previous year (1961).

2 See Michael Healey’s article in this issue for more on how methodological innovations influenced psychiatric understanding.

3 See *Preventing Mental Illness: Past, Present and Future* (Kritsotaki, Long, and Smith 2018) for more historical work on preventive psychiatry.

4 Elizabeth Lunbeck’s *The Americanization of Narcissism* (2014) provides a more positive interpretation of this shift towards focusing on the individual during the 1970s.

5 Although *MHM* was co-authored, the authors of individual chapters were specifically identified. When referencing the book, therefore, I indicate the relevant author/co-authors in text, but refer to all of the authors in references.

6 Yorkville was described as 99% white, whereas Manhattan as a whole was described as 20% non-white, owing to the ethnic make-up of Harlem (Srole et al. 1962:24, 78). Race and US psychiatry has been discussed in depth and from many intriguing perspectives by Doyle (2016), Mendes (2015) Metzl (2010), Raz (2013), and Summers (2019).

7 Leacock’s papers are in the process of being catalogued at the University of Michigan.

8 It is unfortunate that Rubin and Leacock’s correspondences concerning this dispute are not included in the record, since it leaves us with only Opler’s perspective. However, my point is not to assess who was right or wrong, but rather to explore how these tensions affected Midtown’s impact.

9 Rennie also hired Eleanor Leacock despite her Communist Party sympathies. After leaving Midtown, she would continue to be blacklisted from tenured academic positions, working in temporary jobs until being hired by CUNY in 1972 (Price 2004; Wengraf 2008).

10 It directed between 1975 and 2021 by Leighton’s widow, Jane Murphy (1929-2021), and continues to produce research (Lumsden 2006; Gilman et al. 2017).

11 David Paul Lumsden also argues that Leighton was influenced by the approach to public health and mental hygiene espoused by William H. Welch (1850–1934), who died the year Leighton arrived at Johns Hopkins (2006).

12 Indeed, a cordial exchange between Opler and Leighton in January 1956 (prior to Rennie’s death) about the former’s research on Irish ethnicity adds further evidence that this review contributed to the tension (Opler Papers, Box 13, Folder 12).

13 *Pace* Opler, the reviews are not particularly different. While Cluckhohn’s may be more effusive in its praise, Leighton’s review is largely positive.

14 Opler’s name, however, is not associated with any of Volume I’s chapters.

15 Newspaper reports noted how the overshadowing of second volume was ironic, given that *Life Stress and Mental Health* provided insights into what might have driven Lee Harvey Oswald to assassinate Kennedy (Srole Papers, Box 2, Folder 43).

16 Mical Raz has also critiqued how 1960s-era policy makers and mental health experts viewed the parenting skills of low-income parents, especially African-Americans (2013).
